# Bacterial Zoonoses and Infective Endocarditis, Algeria

**DOI:** 10.3201/eid1102.040668

**Published:** 2005-02

**Authors:** Akila Benslimani, Florence Fenollar, Hubert Lepidi, Didier Raoult

**Affiliations:** * Service de Biologie Clinique, Alger, Algérie;; † Université de la Méditerranée, Marseille, France

**Keywords:** endocarditis, diagnosis, polymerase chain reaction, serology, Bartonella quintana, research

## Abstract

Blood culture–negative endocarditis is common in Algeria. We describe the etiology of infective endocarditis in this country. Samples from 110 cases in 108 patients were collected in Algiers. Blood cultures were performed in Algeria. Serologic and molecular analysis of valves was performed in France. Infective endocarditis was classified as definite in 77 cases and possible in 33. Causative agents were detected by blood cultures in 48 cases. All 62 blood culture–negative endocarditis cases were tested by serologic or molecular methods or both. Of these, 34 tested negative and 28 had an etiologic agent identified. A total of 18 infective endocarditis cases were caused by zoonotic and arthropodborne bacteria, including *Bartonella quintana* (14 cases), *Brucella melitensis* (2 cases), and *Coxiella burnetii* (2 cases). Our data underline the high prevalence of infective endocarditis caused by *Bartonella quintana* in northern Africa and the role of serologic and molecular tools for the diagnosis of blood culture–negative endocarditis.

In Algeria, infective endocarditis is common. Vegetations graft primarily on lesions of rheumatic heart disease (*1**,**2*). The rate of blood culture–negative endocarditis in Algeria is as high as 76% (*2*), which leads to difficulty in antimicrobial treatment. Most cases of blood culture–negative endocarditis have been thought to be caused by previous antimicrobial therapy. Infective endocarditis prognosis is often obscured by delayed diagnosis and a lack of specific treatment. In Algeria, poor socioeconomic level and lack of medical follow-up of patients are among the factors associated with endocarditis. The concentration of medical infrastructures in the northern part of the country leads to the referral of patients with serious illnesses, such as endocarditis, to northern hospitals, especially within Algiers (Figure 1). Algiers, the capital and largest city with ≈5 million inhabitants, has 7 hospitals, including 6 cardiology and 5 cardiac surgery wards. These wards receive patients with endocarditis, either for diagnosis and treatment or for corrective surgery of postendocarditis lesions. A retrospective analysis of Algerian infective endocarditis cases showed streptococci and staphylococci were the leading causes, followed by less frequent causes, such as enterobacteria and *Haemophilus* spp (*2*). A high percentage of blood culture–negative endocarditis was noted. However, no study has evaluated the agents responsible for blood culture–negative endocarditis. New serologic and molecular tools, which have improved the etiologic diagnosis of infective endocarditis, have not been used to clarify the unknown role of fastidious bacteria (*3**–**11*). In our study, samples were collected from 110 patients with suspected cases of endocarditis. All samples were analyzed prospectively by using conventional microbiologic methods in Algiers. When available, cardiac valves and serum samples were stored to perform retrospective analysis at the Unité des Rickettsies (Marseille, France).

**Figure 1:**
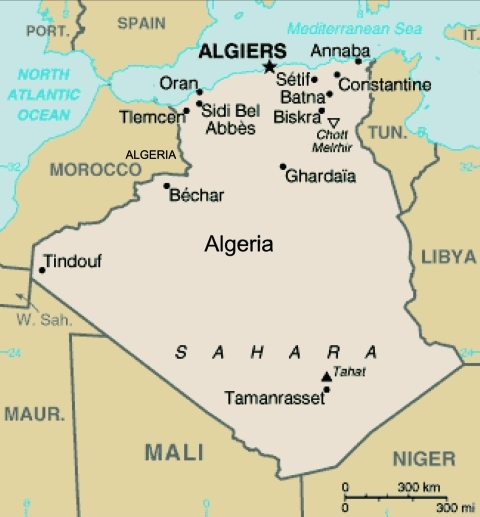
Map of Algeria. Courtesy of Wikipedia Encyclopedia (http://en.wikipedia.org/wiki).

## Material and Methods

### Patients

Clinicians usually diagnose infective endocarditis by using the modified Duke criteria, which includes 3 major criteria (blood cultures typical of infective endocarditis, vegetations on echocardiography, and *Coxiella burnetii* serologic testing with immunoglobulin [Ig] G phase I titer >1:800) and 7 minor criteria (positive blood cultures, fever, previous heart disease, arterial embolism, positive results on serologic examination for endocarditis bacterial pathogens, immunologic disorders, and atypical but compatible findings on echocardiography) (*12*). Definite infective endocarditis is diagnosed if any of the following conditions is met: 2 major criteria exist; 1 major criterion and 3 minor criteria; or 5 minor criteria. Possible infective endocarditis is considered if 1 major criterion and 1 minor criterion or 3 minor criteria exist. On the basis of these criteria, we could locate 110 cases in 108 patients with definite or possible infective endocarditis in 5 cardiology wards and 2 cardiac surgery wards in Algiers during a 42-month period (June 2000–December 2003). For each patient, an information sheet with epidemiologic, clinical, echocardiographic, and biologic data was filled out. A minimum of 3 blood cultures were sampled per patient. Thirty-eight cardiac valve specimens from 38 (35.4%) patients were sampled and stored at –80°C. Thirty-seven cardiac valve specimens from another 30 (27.3%) patients were formalin-fixed for pathologic testing. Sixty-one serum samples from 61 (55.5%) patients were available.

### Blood Cultures

Either Castaneda Aer/Anaer (Bio-Rad, Marnes-La-Coquette, France) or broth for blood culture (Institut Pasteur d'Algérie, Algiers, Algeria) were used as blood-culture medium and were incubated at 37°C. If signs of culture appeared, a blood sample was taken from the culture bottle and Gram staining on Columbia blood agar (BioMérieux, Marcy L'Etoile, France) and chocolate agar (BioMérieux) was performed. Agar plates were incubated in 5% CO_2_ at 37°C. In the event of culture, the microorganism was identified by API identification tests (BioMérieux). At day 15 of incubation, if cultures remained negative, an enrichment of each bottle was processed on Todd-Hewitt broth (Institut Pasteur d'Algérie) supplemented with 0.01% L-cysteine (Sigma-Aldrich, Lausanne, Switzerland) and 0.001% hypochloride pyridoxal (Sigma-Aldrich). In cases of broth turbidity, microscopic examinations were performed as described above. If culture was positive, the strain was identified.

### Valve Analysis

#### Axenic Culture

Thirty-eight excised cardiac valves were examined. If macroscopic lesions of infective endocarditis were detected, we attempted to divide the valve into 3 parts to be used for bacteriologic analysis, storage at –80°C, and histologic analysis. Portions of valve tissue were ground with a mortar and pestle and cultured on Columbia blood agar and chocolate agar supplemented with Polyvitaminic Supplement (Bio-Rad) at 35°C for 15 days in 5% CO_2_. We performed direct Gram staining and identified colonies as described above.

#### Cell Culture

Cell cultures were performed in France. Specimens from 12 cardiac valves positive on polymerase chain reaction (PCR) for *Bartonella quintana* or *Brucella melitensis* were spread onto cells grown within a shell vial as previously described (*13**,**14*). After 3 weeks of incubation at 37°C, the bacteria were detected by using Gimenez staining, a direct immunofluorescence test incorporating polyclonal antibodies directed against *Bartonella*, and by PCR targeting the 16S rRNA sequence.

#### Molecular Biology

For the 38 cardiac samples stored at –80°C, molecular analysis was performed in France. After 18 hours of proteinase K digestion at 55°C, DNA was extracted from tissue by using the MagNA Pure LC instrument (Roche Molecular Biochemicals, Manheim, Germany) and MagNA Pure LC DNA Isolation Kit III (Roche Molecular Biochemicals), as described by the manufacturer. A PCR-positive valve sample taken from a patient with *Staphylococcus aureus* endocarditis was used as a positive control. A mixture of all reagents used for DNA extraction and DNA extracted from normal heart tissue were processed as negative controls. One negative control was included for every 5 samples tested. PCR amplification and sequencing were performed, as previously described (*15*), by using primers in Table 1. PCR targeting the 16S rRNA sequence was systematically performed. When a negative result occurred, additional PCR was performed targeting the 18S and 28S rRNA internal transcribed spacer to search for fungal infections. All positive PCR products were sequenced. The sequences were compared to those available in GenBank. Positive PCR results were considered as certain, when congruence existed between the results obtained with PCR and those obtained with other analyses. With a positive result interpreted as a possible case, we performed additional PCR, targeting a second gene with genus-specific primers (Table 1). When the PCR was positive and the sequence gave the same result, the case was reclassified as certain. When the second PCR was negative, we performed a PCR targeting a third gene. When both PCRs targeting the second and the third gene were negative, the result was classified as negative.

**Table 1:** Primers used for broad-range 16S rRNA polymerase chain reaction (PCR) and, according to species identified by sequencing, primers targeting a second gene for confirmation of positive 16S rRNA PCR results and primers used for fungal PCR

Microorganisms	Gene	Forward primer	Reverse primer
Eubacteria	16S rRNA	536f 5´ CAGCAGCCGCGGTAATAC	RP2 5´ ACGGCTACCTTGTTACGACTT
*Staphylococcus* spp.	RpoB	StphF 5´ AAACCIATACGCAATTGGTT	StphR 5´ GTTTCATGACTTGGGACGG
*Streptococcus* spp.	RpoB	StrpF 5´ AARYTIGGMCCTGAAGAAAT	StrpR 5´ TGIARTTTRTCATCAACCATGTG
*Enterococcus* spp.	RpoB	StrpF 5´ AARYTIGGMCCTGAAGAAAT	StrpR 5´ TGIARTTTRTCATCAACCATGTG
*Streptococcus* spp.	*SOD*	d1 5´ CCITAYICITAYGAYGCIYTIGARCC	d2 5´ ARRTARTAIGCRTGYTCCCAIACRTC
*Enterobacteriaceae*	RpoB	CM7 5´ AACCAGTTCCGCGTTGGCCTGG	CM31b 5´ CCTGAACAACACGCTCGGA
*Mycoplasma hominis*	*FtsY*	MH1F 5´ GTGTTGTATCGACAACAG	MH1R 5´ GTGTTGTATCGACAA
*Coxiella burnetii*	IS111	Trans3 5´ CAACTGTGTGGAATTGATGA	Trans5 5´ TTTACATGACGCAATAGCGC
*Bartonella* spp.	ITS	ITSF1 5´ GCGACTGGGGTGAAGTGG	ITSR1 5´ AGGCTTGGGATCATCATC
*Bacillus* spp.	*RpoB*	Bc55F 5´ TCTCGTATGGAACGTGTTGT	Bc260R 5´ TGAACGTCACGYACTTCAAA
*Corynebacterium* spp.	*RpoB*	C2700F 5´ GWATGAACATYGGBCAGGT	C3130R 5´ TCCATYTCRCCRAARCGCT
Fungi	18S-28S ITS	FCU 5´ TCCGTAGGTGAACCTGCGG	RCU 5´ GCTGCGTTCTTCATCGATGC

#### Histologic and Immunohistologic Analysis

Thirty-seven valve samples underwent fixation by formalin and were paraffin-embedded. Valve specimens were cut to 3-mm thickness serial sections. Hematoxylin-eosin-saffron, periodic acid-Schiff, Giemsa, Brown-Hopps/Brown-Brenn Gram, Grocott-Gomori methenamine silver, and Warthin-Starry stains were used (*16*). On the basis of the histologic findings, valve specimens were divided into 3 groups: A, B, and C. Group A samples showed histologic features of infective endocarditis consisting of vegetations or polymorphonuclear leukocyte–rich valvular inflammation. Group B specimens showed valvular inflammation composed of mainly inflammatory mononuclear cells, macrophages, and lymphocytes without vegetations and microorganisms. Group C samples were devoid of inflammation, vegetations, or microorganisms. When *Bartonella* endocarditis was suspected, immunohistochemical analysis was performed on valve sections with an anti-*Bartonella* rabbit polyclonal antibody as previously described (*17*).

### Serum Sample Analysis

#### Serologic Testing

*Brucella* serologic analysis was performed by Rose-Bengale agglutination (Bio-Rad, Marnes-La-Coquette, France) for 61 serum samples from 61 patients in Algiers, and the samples were stored at –20°C for further study. The confirmation was observed by Wright Serology (Bio-Rad). In the case of endocarditis, specific antibody titers exceeded 1:800. *Bartonella* and *C. burnetii* serologic testing was performed in France on all 61 samples. For *Bartonella* serologic testing, *B. quintana* and *B. henselae* were used as antigens in a microimmunofluorescence (MIF) assay performed as previously described (*18*). A patient was considered to have *Bartonella* endocarditis when IgG titers >1:800 were observed (*18*). The species identification was performed with Western blot performed before and after serum cross-adsorption as previously described (*19*). For *C. burnetii* serologic testing, immunoglobulin (Ig) G, IgM, and IgA antibody titers were estimated by using an MIF test as previously described (*20*). A diagnosis of chronic endocarditis was made when a patient had an IgG phase I titer >1:800 (*20*). A LightCycler nested PCR was performed on positive serum samples for *Bartonella* and *C. burnetii* as previously described (*21**,**22*).

## Results

### Patient Characteristics

Our prospective study led to identification of 110 cases from 108 patients. The 110 episodes were classified as 77 (70%) definite infective endocarditis and 33 (30%) possible infective endocarditis (*12*). A second episode of infective endocarditis developed in 2 patients during our survey. The patients included 64 men and 40 women with a mean age of 35.3 years (range 17–72 years). The series included 4 children, 2 boys (6 and 8 years of age) and 2 girls (9 and 14 years of age). Among the patients, 34 came from rural areas, 61 lived in urban areas, 1 was in prison, and no information could be obtained for 12. Among 96 patients whose living conditions were known, 59 (61.5%) lived in poor and crowded families of at least 10 persons. Among the 110 cases, 87 (79%) episodes were diagnosed on native valve and 23 (21%) on prosthetic valve. The mitral valve was affected in 31 (28.2%) cases, the aortic in 29 (26.3%), and both in 41 (37.2%). The tricuspid valve was affected in 3 (2.7%) patients, and 4 (3.6%) had aortic, mitral, and tricuspid involvement. We reported 1 case with mitral and pulmonary valves affected, with the persistence of an arterial canal, and 1 patient on a pacemaker.

### Blood Cultures

Blood cultures identified 48 microorganisms (Table 2). Of the 22 *Streptococcus* spp. cultures, 5 *Streptococcus mitis*, 6 *Streptococcus* sp., 3 *S. agalactiae*, 3 *Granulicatella adiacens*, 2 a-*Streptococcus*, 1 *S. oralis*, 1 *S. intermedius*, and 1 *Gemella morbillorum* were identified. Seven *Staphylococcus aureus* and 5 coagulase-negative *Staphylococcus* were observed. One *Haemophilus influenzae*, 1 *H. aphrophilus*, 1 *Haemophilus* sp., 1 *Kingella kingae*, and 1 *Actinobacillus actinomycetemcomitans* were identified among the HACEK group (*Haemophilus*, *Actinobacillus*, *Cardiobacterium*, *Eikenella*, *Kingella*). One *Brucella melitensis*, a zoonotic agent, was isolated.

**Table 2:** Distribution of 110 infective endocarditis cases* diagnosed in Algeria using blood culture, cardiac valve culture, serologic testing, cardiac valve polymerase chain reaction (PCR), and PCR on serum samples

Identified microorganisms	Positive samples/tested samples					
	Blood culture (N = 110)	Cardiac valve culture (N = 38)	Serologic testing (N = 61)	Cardiac valve PCR (N = 38)	PCR on serum sample (N = 9)	Total
*Streptococcus* spp. and related genera	0/22	0/4	NP	7/0	NP†	24/0
*Bartonella quintana*	0/1‡	0/3	5/2	10/0	3/2	12/2
*Staphylococcus* spp.	2/10	0/3	NP	2/1	NP	11/3
HACEK§	0/4	0/0	NP	1/1	NP	5/1
*Enterococcus* spp.	1/1	0/0	NP	1/0	NP	2/1
*Brucella melitensis*	0/1	0/0	2/0	2/0	NP	2/0
*Coxiella burnetii*	0/0	0/0	2/0	0/0	1/NP	2/0
*Corynebacterium* spp.	0/2	0/1	NP	1/0	NP	2/0
*Mycoplasma homini*s	0/0	0/0	NP	1/0	NP	1/0
Enterobacteria spp.	1/1	0/0	NP	0/0	NP	1/1
*Alcaligenes faecalis*	0/1	0/0	NP	0/0	NP	1/0
*Pseudomonas aeruginosa*	0/1	0/0	NP	0/0	NP	1/0
*Bacillus cereus*	0/0	0/0	NP	1/0	NP	1/0
*Candida* spp.	0/0	0/1	NP	1/0	NP	1/0
	Negative samples for definite infective endocarditis/negative samples for possible infective endocarditis					
No etiology	10/25	2/7	8/20	2/7	NP/NP	

*77 definite and 33 possible. †NP, not performed. ‡If we consider that *Bartonella quintana* was misidentified as *Haemophilus influenzae*. §HACEK, *Haemophilus*, *Actinobacillus*, *Cardiobacterium*, *Eikenella*, *Kingella*.

### Serum Analysis

**Figure 2:**
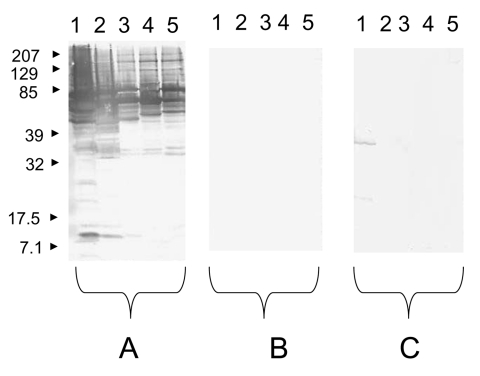
Western blot performed with a serum sample from a patient with an endocarditis caused by *Bartonella quintana*. Molecular masses (in kilodaltons) are given to the left of the panels. A) Untreated serum sample analyzed with *B. quintana* (lane 1), *B. henselae* (lane 2), *B. elizabethae* (lane 3), *B. vinsonii* subsp. *arupensis* (lane 4), and *B. vinsonii* subsp. *berkhoffii* (lane 5) antigens. B) *B. quintana*–adsorbed serum sample analyzed with *B. quintana* (lane 1), *B. henselae* (lane 2), *B. elizabethae* (lane 3), *B. vinsonii* subsp. *arupensis* (lane 4), and *B. vinsonii* subsp. *berkhoffii* (lane 5) antigens. C) *B. henselae*–adsorbed serum analyzed with *B. quintana* (lane 1), *B. henselae* (lane 2), *B. elizabethae* (lane 3), *B. vinsonii* subsp. *arupensis* (lane 4), and *B. vinsonii* subsp. *berkhoffii* (lane 5) antigens.

Using serologic testing, infective endocarditis could be diagnosed in 11 (18%) of 61 serum samples. A positive *Brucella* serologic result with titers of 1:3,200 was observed for 2 patients (1 sample was also culture positive). Two other patients had a typical profile of Q fever endocarditis (Phase I: IgG 1:3,200; IgM 1:25; IgA 1:1,600/Phase II: IgG 1:6,400; IgM 1:25; IgA 1:1,600 for 1 patient and Phase I: IgG 1:6,400; IgM 1:800; IgA 1:50/Phase II: IgG 1:12,800; IgM 1:800; IgA 1:100 for the other patient). Among these 2 patients, *C. burnetii* LightCycler nested-PCR performed on serum samples was positive for the sample from 1 patient. A positive *Bartonella* serologic result, with IgG >1:800, was observed for 7 patients (Table 3). The Western-blot analysis of the 7 serum samples allowed the specific diagnosis of *B. quintana* (Figure 2). Of these 7 patients, *B. quintana* LightCycler nested-PCR performed on serum samples was positive for 5 patients (Table 3).

**Table 3:** Living conditions, involved cardiac valves, and diagnostic tools for *Bartonella quintana* endocarditis cases in 14 patients*

Patient	Living conditions	Involved cardiac valves	Blood culture	*Bartonella* serologic testing	PCR on serum sample	Cardiac valve culture	Cardiac valve PCR	Histologic analysis
1	Poor rural area	Aortic	–	1:800	*B. quintana*	*B. quintana*	*B. quintana*	NP
2	Poor rural area	Mitral	–	1:1,600	–	–	*B. quintana*	WS+/IC+
3	Poor urban area	Mitral	–	1:800	*B. quintana*	NP	NP	WS+/IC+
4	Poor rural area	Aortic	–	NP	NP	–	*B. quintana*	NP
5	Poor urban area	Tricuspid	+†	1:1,600	*B. quintana*	NP	NP	NP
6	Poor rural area	Aortic + mitral	–	NP	NP	–	*B. quintana*	WS+/IC+
7	Unknown	Aortic + mitral	–	NP	NP	–	*B. quintana*	WS+/IC+
8	Poor urban area	Aortic	–	1:800	*B. quintana*	–	*B. quintana*	WS+/IC+
9	Good urban area	Aortic	–	NP	NP	*B. quintana*	*B. quintana*	WS+/IC+
10	Good rural area	Aortic + mitral	–	NP	NP	*B. quintana*	*B. quintana*	WS+/IC+
11	Poor urban area	Mitral	–	1:3,200	–	NP	NP	NP
12	Poor rural area	Aortic	–	1:3,200	*B. quintana*	NP	NP	NP
13	Poor rural area	Aortic	–	NP	NP	–	*B. quintana*	NP
14	Poor rural area	Aortic	–	NP	NP	–	*B. quintana*	NP

*NP, not performed; WS+, Warthin-Starry positive; IC+, immunochemistry positive; PCR, polymerase chain reaction. †If we consider that *B. quintana* was misidentified as *Haemophilus influenzae*.

### Cardiac Valve Analysis

**Figure 3:**
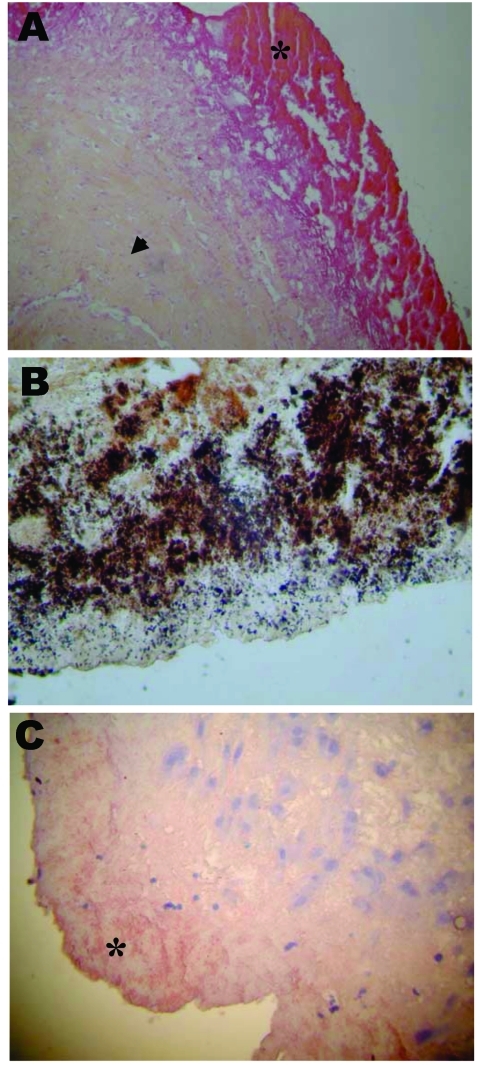
A) Section of an aortic valve from a patient with *Bartonella* endocarditis. Note the extensive fibrosis of the connective valve tissue (arrowhead), the vegetation (*), and the low inflammatory infiltrate of the valve tissue (hematoxylin-phloxine-saffron, original magnification 100x). B) Resected valve with *Bartonella quintana* infection showing darkly stained bacilli consistent with *Bartonella*. Note the numerous clusters of argyrophilic bacteria present in the valvular vegetation (Warthin-Starry silver, original magnification 1,000x). C) Immunohistochemical detection of *B. quintana* in a resected valve from a patient with *Bartonella* endocarditis. Note the extracellular distribution of the bacterial colonies (*) in the valvular vegetation (polyclonal antibody and hematoxylin counterstain, original magnification 250x).

Axenic culture of cardiac valves was positive for 9 samples. The growth of 2 coagulase-negative *Staphylococcus*, 2 *Streptococcus* sp., 1 *Staphylococcus aureus*, 1 *Streptococcus mitis*, 1 *S. intermedius*, 1 *Corynebacterium* sp., and 1 *Candida kruzei* was observed. Another sample was polymicrobial. Cell culture allowed the growth of *B. quintana*, an arthropodborne disease agent, from 3 valve samples (Tables 2 and 3). The numbers of valve specimens classified into groups A, B, and C were 21, 5, and 11, respectively. With the exception of *Bartonella* endocarditis, the samples with histologic features of infective endocarditis had vegetations in most cases, moderate fibrosis, calcifications in some cases, and numerous inflammatory infiltrates composed predominantly of polymorphonuclear leukocytes and abundant neovascularization. By using special stains, microorganisms were visualized in 16 samples from group A, gram-positive cocci and gram-negative bacilli in 8 cases each. In samples from group B, the inflammatory infiltrates were rare and focal and consisted mainly of macrophages and lymphocytes with discrete neovascularization. The specimens from group C showed noninflammatory degenerative damage with extensive fibrosis and often calcifications. The histologic features of *Bartonella* endocarditis were different from the other infective endocarditis. Samples from 7 cases with *Bartonella* endocarditis were examined. The valve tissues showed degenerative damage with extensive fibrosis. The valve tissues were poorly inflamed with rare mononuclear inflammatory cell infiltrates composed of lymphocytes and macrophages and discrete neovascularization. Vegetations, present in all samples, were small in size. In all cases, the Warthin-Starry stain detected *Bartonella*, mainly in vegetations as small bacillary organisms (Figure 3).

The 16S rRNA PCR was positive for 29 cardiac valves (Tables 2 and 4). *B. quintana* was detected on 10 specimens (Table 3). Among the *Streptococcus* spp. and related genera, 3 *Streptococcus* sp., 1 *S. mitis*, 1 *S. mutans*, 1 *S. gordonii*, 1 *S. pneumoniae*, and 1 *Granulicatella adiacens* were detected. Two *Staphylococcus aureus* and 1 coagulase-negative *Staphylococcus* were identified. Among the 2 bacteria from the HACEK group, 1 *H. paraphrophilus* and 1 *Cardiobacterium hominis* were identified. PCR performed with a second gene confirmed the previous PCR results with 1 exception. One *Streptococcus* sp. was not retrieved by PCR targeting a second or third gene and was considered as contamination. The PCR targeting the 18S–28S rRNA ITS allowed the detection of 1 *Candida parapsilosis*. Finally, *Bartonella* spp. were also specifically visualized in vegetations by immunohistochemistry in all the cases of *B. quintana* endocarditis (Figure 3).

**Table 4:** Discrepant results between blood culture, cardiac valve culture, cardiac valve PCR, and serologic testing for 9 patients*

Patient	Blood culture	Cardiac valve culture	16S rRNA PCR	PCR targeting another gene	Histologic analysis	Serologic testing	Conclusions
1†	Negative	*Candida krusei*	*Streptococcus* spp.	*Rpo*B: negative SOD: negative 18-28S ITS: *C. parapsilosis*	NP	Negative	*C. parapsilosis*
2‡	Negative	Polymicrobial	*Streptococcus mitis*	*S. mitis*	A	Negative	*S. mitis*
3‡	Negative	CNS	*Haemophilus paraphrophilus*	NP	A / BGN	Negative	*H. paraphrophilus*
4‡	Negative	CNS	*Bartonella quintana*	*B. quintana*	NP	NP	*B. quintana*
5‡	*S. mitis*	*Staphylococcus aureus*	*Streptococcus gordonii*	*S. gordonii*	A / CGP	Negative	*S. gordonii*
6†	*H. influenzae*	NP	NP	NP	NP	1:1,600 Positive PCR on serum samples	*B. quintana*
7†	*Streptococcus intermedius*	*Streptococcus intermedius*	*S. mutans*	*S. mutans*	A	NP	*S. mutans*
8‡	Negative	*Corynebacterium* spp.	*Bacillus cereus*	*B. cereus*	A	Negative	*B. cereus*
9‡	Negative	*S. mitis*	*Enterococcus gallinarum*	*E. gallinarum*	A / CGP	Negative	*E. gallinarum*

*PCR, polymerase chain reaction; NP, not performed; CNS, coagulase-negative *Staphylococcus*; BGN, bacillus gram negative; CGP, cocci gram positive. †The microorganisms detected in valve culture were contaminants. ‡The microorganisms were misidentified.

### Causative Microorganisms and Discordant Results

The overall distribution of causative microorganisms and their identification, depending on the diagnostic tools used, are displayed in Table 2. An etiologic agent could not be determined for 10 (13%) of definite cases and 28 (76%) of possible cases. For the 2 patients with recurring infective endocarditis, the cause for the first episode was different than that of the second episode. One patient had endocarditis caused by *Streptococcus oralis*, and 1 year later, endocarditis caused by *K. kingae* developed. For the other patient, no etiologic diagnosis was established for the first episode, during which a valve removal was necessary. Four months after cardiac surgery, the patient had endocarditis caused by *Staphylococcus epidermidis*. Nine discrepant results were also observed and are summarized in Table 4.

## Discussion

Endocarditis cases with fastidious agents escape microbiologic diagnosis classically applied in Algerian laboratories. For the first time, we established a profile of the microbiologic etiology of infective endocarditis in Algeria. Our conclusions concerning PCR results were submitted to a rigorous strategy of validation. All of the controls must be correct for validating each assay. The result was considered true if confirmation was obtained by successfully amplifying bacterial DNA when targeting another gene, the PCR result was congruent with the results of other diagnostic tools, or both.

Of the 77 cases of definite infective endocarditis, the cause was found for 67 (87%) cases. The diagnosis was performed on the basis of positive blood cultures for 44 cases. For 20 (26%) cases, no etiologic diagnosis was obtained in Algeria but was performed in France on the basis of cardiac valve PCR, and *Bartonella* and *Coxiella burnetii* serologic testing. These data show improvement in the etiologic diagnosis of endocarditis when molecular or serologic tools are used. The rate of remaining infective endocarditis without cause is comparable to the prevalence in western countries (*16*). As in other countries, the etiologic distribution is dominated by the bacteria responsible for infective endocarditis, such as *Streptococcus* spp. and related genera, *Staphylococcus* spp., and bacteria from the HACEK group. The difference in comparison to other countries is that blood culture–negative endocarditis is mainly linked to zoonotic and arthropodborne agents.

For the 33 cases of possible infective endocarditis, the number of etiologic diagnoses was fewer than those for definitive infective endocarditis However, in this group, some cases are infective endocarditis and others are not. If we consider a *Bartonella* serologic result >1:800 as a major criterion (*5*), the 2 possible cases of *B. quintana* infective endocarditis will be classified as definite. Therefore, *Bartonella* serologic results should be taken into account in future revisions of the Duke criteria. Of the 48 case-patients with positive blood cultures, 19 had additional samples tested through a second analysis (serologic or molecular methods). Of the 19, 11 had negative results, 5 were concordant, and 3 were discordant. Of these 48 cultures, 1 corresponds to brucellosis.

Of the 62 blood culture–negative endocarditis cases, samples from all were tested by serologic or molecular methods. Of these, 34 were negative, and 28 had an etiologic agent identified. Seventeen of those were due to zoonoses or arthropodborne bacterial diseases.

Discrepancies were observed between the results obtained by using the various techniques. Some discrepancies resulted from culture contamination with the cutaneous flora. A significant rate of contamination has been already reported, and the low specificity of valve culture that we observed confirms these results (*23**–**25*). One discrepancy was caused by identification problems at the species level for *Streptococcus*. This fact has been previously reported (*7*). Another discrepancy was linked to a *Candida* species misidentification by phenotypic analysis, which was corrected by using molecular tools. The last discordant case corresponded to a patient for whom blood cultures were positive for *H. influenzae*. When serum samples were analyzed, a diagnosis of *B. quintana* endocarditis has been established in the presence of positive *Bartonella* MIF, Western blot, and PCR. We do not know if *B. quintana* was misidentified as *H. influenzae*, which is possible as both are slow-growing, hemin-dependent, small, gram-negative bacteria (*26*). We believe that as fastidious, small, gram-negative bacteria growing in blood agar, the 2 organisms may be confused.

In Algeria, cases of infective endocarditis caused by zoonotic and arthropodborne disease agents, such as *Coxiella burnetii*, *Brucella melitensis*, and *Bartonella quintana* are frequently observed and correspond to one quarter of the performed diagnoses. *B. quintana* would be one of the most common agents of infective endocarditis in our Algiers series (15.6% of definite infective endocarditis). The prevalence of endocarditis caused by *Bartonella* varies depends on the country. In Canada, *Bartonella* causes 3% of endocarditis cases (*27*). In Sweden, no *Bartonella* endocarditis was identified in an analysis of 334 infective endocarditis cases (*28*). In the United Kingdom, *Bartonella* endocarditis accounts for 1.1% of infective endocarditis cases (*29*). In Germany and in France, *Bartonella* endocarditis accounts for 3% of all infective endocarditis (A. Sander et al. unpub. data) (*27*). The frequency of *Bartonella* endocarditis is <1% for Sweden and higher in France, Germany, the United Kingdom (3%), and North Africa (15%). Such differences may be linked to differences in living conditions.

Homeless people are at risk for *B. quintana* endocarditis (*30**,**31*). Indeed, *B. quintana*, like *Rickettsia prowazekii*, the agent of epidemic typhus, is transmitted by body lice. Those who live in extreme poverty are often the persons who are infested. The recent description of typhus in Algeria confirms that poor socioeconomic conditions still exist in this country (*32**–**34*). In our studies, *B. quintana* endocarditis cases occurred in patients living in poor conditions. Although the only known reservoir for *B. quintana* is humans, the bacterium has recently been associated with fleas (*35*). Moreover, some cases of *B. quintana* infections have been linked to contact with cats and cat fleas in patients who were not homeless and did not have body lice (*36*).

*Brucella melitensis*, well known in northern Africa, where bucellosis is endemic in certain areas, accounts for 2.6% of all infective endocarditis cases for which an etiologic diagnosis has been performed (*37*). Be cause *C. burnetii* detection requires specialized tests not normally found in most laboratories, it is not often diagnosed in Algeria (*38*). Two cases were retrospectively detected.

Importance of infective endocarditis caused by zoonotic and arthropodborne agents in Algeria leads to 2 considerations. First, specific serologic tests need to be used for diagnosis. Indeed, 25% of our etiologic diagnoses correspond to microorganisms for which the diagnosis is usually based on serologic testing. Secondly, the therapeutic impact of *Brucella* and *Coxiella* diagnosis is important because the antimicrobial treatment of endocarditis caused by these agents must include doxycycline. The 2 patients with Q fever endocarditis died during their hospitalization because of inadequate antimicrobial therapy. Finally, the high rate of blood culture–negative endocarditis was not linked to prior antimicrobial therapy but rather to fastidious microorganisms for which serologic testing (as for zoonotic and arthropodborne disease agents) or molecular analysis (as for *Mycoplasma hominis* [39] and *Corynebacterium* spp.) are diagnostic tools.

Our study underlines the need to perform serologic analysis to determine for the etiology of infective endocarditis. *Bartonella* serologic testing is an important tool for diagnosis of blood culture–negative endocarditis and should be taken into account in future revisions of the Duke criteria. This study made it possible to show that zoonotic and arthropodborne disease agents cause one quarter of infective endocarditis in Algeria; *B. quintana* caused 13% of our cases.
